# Prognostic value of tumor length and diameter for esophageal squamous cell cancer patients treated with definitive (chemo)radiotherapy: Potential indicators for nonsurgical T staging

**DOI:** 10.1002/cam4.2532

**Published:** 2019-09-04

**Authors:** Hongyao Xu, Shengxi Wu, Hesan Luo, Chuyun Chen, Lianxing Lin, Hecheng Huang, Renliang Xue

**Affiliations:** ^1^ Department of Radiation Oncology Shantou Central Hospital Affiliated Shantou Hospital of Sun Yat‐sen University Shantou China

**Keywords:** esophageal squamous cell cancer, neoplasm staging, radiotherapy, tumor diameter, tumor length

## Abstract

**Purpose:**

The aim of this work was to evaluate the prognostic value of tumor length and diameter for patients with esophageal squamous cell cancer (ESCC) treated with definitive (chemo)radiotherapy to identify potential indicators for separate nonsurgical T staging, which are needed in clinical practice.

**Materials and Methods:**

A total of 682 patients with ESCC who underwent definitive (chemo)radiotherapy between 2009 and 2015 were reviewed. Esophageal tumor length and diameter were determined by barium esophagography and computed tomography before treatment. Univariate and multivariate analyses were used to assess the impact of tumor length and diameter on long‐term overall survival (OS) and progression‐free survival (PFS). Propensity score matching (PSM) analysis was also used to control intergroup heterogeneity.

**Results:**

The median OS and PFS were 22.2 months and 15.4 months, respectively, in the tumor length ≤ 6 cm group, which were significantly longer than those in the tumor length > 6 cm group (13.4 and 8.5 months, respectively). The median OS and PFS were 23.3 months and 15.9 months, respectively, in the tumor diameter ≤ 3.5 cm group, which were also significantly longer than those in the tumor diameter > 3.5 cm group (13.3 and 8.8 months, respectively). Similar results were found after PSM. Univariate and multivariate analyses showed that tumor length and diameter were both independent predictors of long‐term survival.

**Conclusion:**

Tumor length and diameter are both independent prognostic factors for ESCC patients treated with definitive (chemo)radiotherapy. These two imaging parameters have the potential for development and use in nonsurgical T staging.

## Graphical abstract

Tumor length and diameter are both independent prognostic factors for esophageal squamous cell cancer patients treated with definitive (chemo)radiotherapy. These two imaging parameters have the potential to develop nonsurgical T staging.

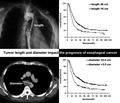

## 1 INTRODUCTION

Esophageal cancer was the seventh most frequent malignancy and the sixth leading cause of cancer death worldwide in 2018. Eastern Asia has the highest incidence of esophageal cancer, and over 90% of the cases in this region were diagnosed with squamous cell cancer.^1^

Currently, definitive (chemo)radiotherapy is considered the primary treatment for patients with locally unresectable disease and those who are medically inoperable or hope to preserve their esophagus.^2^ However, the current American Joint Committee on Cancer TNM staging system for esophageal cancer was driven by the survival data of patients who received esophagectomy as a major treatment; therefore, its guiding significance and predictive value for those who receive (chemo)radiation are limited.^3^, ^4^ Moreover, the current staging system shares pathological parameters that are difficult to determine without surgery. Modern imaging techniques can somehow reflect these pathological parameters but are limited by the resolution of each individual modality.^5^ Using histologic criteria but based largely on imaging makes this staging coarse in nature.^3^ Hence, it is reasonable to develop a nonsurgical TNM staging system that is more practical and feasible for esophageal cancer, especially for patients mainly treated with radiation.

With this purpose in mind, potential indicators for nonsurgical T staging were preliminarily investigated in this study. The present T category depends on the depth of invasion into the esophageal wall, which can be subdivided into the submucosa, muscularis propria, and adventitia. The difficulty in distinguishing these anatomical layers usually causes error in clinical T staging.^6^, ^7^ At present, endoscopic ultrasound (EUS) is considered the optimal imaging technique to differentiate the T category, with accuracy typically ranging from 70% to 90%.^5^, ^7^ However, some studies have reported that the rate of EUS errors in predicting T stage was as high as approximately 50%, especially in tumors with longer lengths.^6^, ^7^ Even in T1‐ and T2‐stage esophageal cancer, T staging by EUS showed less than a moderate degree of agreement with pathologic T staging.^8^ Additionally, EUS has other disadvantages that should not be ignored. First, it is an invasive technique with potential risks, such as esophageal perforation, hemorrhage and complications associated with sedation. Second, the failure to cross stenotic tumors should be noted, which was reported to occur in 30% of cases.^5^ Third, due to cost constraints and the regional availability of staging modalities, EUS cannot be used routinely worldwide. Therefore, we hope to use other prognostic parameters that can be easily accessed to guide the T staging of esophageal cancer.

According to other solid tumor staging criteria, such as those for lung and breast cancer, the greatest dimension of the primary tumor should be taken into account. For esophageal cancer, it can be interpreted as the longitudinal length or the transverse diameter. Specifically, for patients undergoing radiotherapy, a larger tumor diameter or longer length may indicate greater treatment toxicity because the radiation area is therefore larger. In fact, tumor length was used as a criterion for esophageal cancer staging in the past but was abandoned in 1987.^9^ However, increasing evidence has shown that tumor length greatly affects patient prognosis, and researchers have recommended adding it to staging.^10^, ^11^, ^12^, ^13^, ^14^, ^15^, ^16^ Tumor length seems to be a promising indicator, but controversy still exists regarding the use of this parameter.^17^, ^18^, ^19^ On the other hand, some researchers have found that the esophageal tumor diameter is an independent factor for survival.^18^, ^20^ Replacing relative depth with absolute diameter sounds difficult but may be feasible because they are on the same dimension. Hence, in this study, we targeted patients with esophageal squamous cell cancer (ESCC) treated with definitive (chemo)radiotherapy and aimed to identify whether tumor length and diameter could predict prognosis and serve as potential T staging indicators.

## 2 METHODS

### 2.1 Patients and pretreatment evaluation

Consecutive ESCC patients who underwent definitive (chemo)radiotherapy with curative intent at our hospital between January 2009 and December 2015 were retrospectively analyzed. Patients with other pathological types, distant metastasis, a prior or concomitant malignancy, and esophageal duplicate cancer and those with incomplete records were excluded. A pretreatment evaluation included standard laboratory tests, a physical examination, barium esophagography, cervical/chest/abdominal computed tomography (CT) with intravenous contrast and upper gastrointestinal endoscopy. EUS and PET/CT were performed on a portion of patients. Clinical staging was performed according to the 8th edition of TNM staging for esophageal cancer. Tumor length was determined by barium esophagography according to institutional practice guidelines, and tumor diameter was determined by the maximum esophageal diameter shown by CT.

This study was carried out in accordance with the principles of the Declaration of Helsinki. The Institutional Review Board of our hospital approved this study and waived the requirement for written informed consent due to its retrospective nature.

### 2.2 Treatment

All patients underwent a CT or PET/CT scan, and 3‐dimensional images were then reconstructed in the treatment planning system. External irradiation was performed with a 6 MV X‐ray linear accelerator. All patients were treated with three‐dimensional conformal radiotherapy or intensity‐modulated radiation. The target areas were evaluated by at least two radiologists, and any discrepancy was resolved by discussion. Determining the gross tumor volume (GTV) involves imaging positive lesions; GTV‐N includes the clinical diagnosis of positive lymph nodes (supraclavicular node with a short diameter (>5 mm)^21^ and the mediastinal lymph node above the tracheal fork with a short diameter (>5 mm), while other lymph nodes have a diameter > 10 mm^22^). The clinical target volume (CTV) was contoured based on the GTV and GTV‐N, with an external expansion of 3 cm (up and down directions) and 0.5 cm (anterior and posterior; left and right directions). The CTV of the upper thoracic EC includes the bilateral supraclavicular region. Radiation was delivered with the following normal tissue constraints: <30% volume of the lungs receiving 20 Gy; <50% volume of the heart receiving 45 Gy; and < 10% volume of the spinal cord receiving 50 Gy. Chemotherapy was administered as a concurrent and/or adjuvant schedule for most patients. Concurrent chemotherapy mainly consisted of weekly docetaxel, cisplatin or nedaplatin (25 mg/m^2^) targeted at five to six courses in total. Adjuvant chemotherapy consisted of two to four cycles of platinum‐based chemotherapy (20‐25 mg/m^2^, days 1‐3) with 5‐FU (750 mg/m^2^, days 1‐4) or docetaxel (75 mg/m^2^, day 1) every 28 days.

### 2.3 Statistical analysis

The Kaplan–Meier method was used to analyze survival, and intergroup differences were examined using the log‐rank test. A progression‐free event was defined as the first documented radiographic evidence of progressive disease or death from any cause. Univariate analysis was used to estimate the prognostic significance of potential parameters. Variables that significantly affected survival were then included in the multivariate model. Multivariate analysis was performed using Cox regression to evaluate independent prognostic factors associated with overall survival (OS) and progression‐free survival (PFS), and a p‐value of *P* < .05 was considered statistically significant. The optimal cut‐off values for tumor length and diameter as prognostic variables were chosen based on the median and referred to a receiver operating characteristic (ROC) curve analysis with ‘tumor length (or diameter)’ as the criterion variable and ‘progress’ as the condition variable. Propensity score matching (PSM) analysis (including variables such as age, sex, tumor location, nodal status and chemotherapy) was performed using the one‐to‐one nearest neighbor method. Statistical analyses were performed using the Statistical Package for the Social Sciences (SPSS, version 22.0).

## 3 RESULTS

### 3.1 Patient characteristics

A total of 682 eligible patients with ESCC were included in the study. In general, approximately half of the patients were older than 65 years, and 496 (73%) patients were male. More than half of the primary tumors were located in the middle thoracic portion, while only 13% were located in the lower esophagus. Approximately, one‐third of patients refused chemotherapy or were medically intolerant; therefore, they received definitive radiotherapy alone. The median radiotherapy dose was 64 Gy (46‐70 Gy). The median follow‐up duration for living patients was 57.5 months (range 25.2‐112.5 months). Only 2 cases were lost to follow‐up and were defined as censored cases.

Among the 682 patients, the tumor length ranged from 1.0 to 18 cm (mean, 6.12 cm; median, 6.0 cm), and the tumor diameter ranged from 1.4 to 6.6 cm (mean, 3.56 cm; median, 3.5 cm). The results of ROC analysis revealed that a median tumor length of 6.0 cm and a tumor diameter of 3.5 cm were suitable threshold values due to their high sensitivity and specificity (Table S1). Therefore, 6.0 cm for tumor length and 3.5 cm for tumor diameter were defined as the cut‐off values for further analysis. Patient characteristics according to tumor length and diameter are shown in Tables 1 and 2, respectively. Statistically significant differences were observed between the length groups with respect to age, sex, tumor location, and N stage. Significant differences were also found between the diameter groups with respect to sex, location, N stage and chemotherapy (nearly statistically significant). After PSM, 212 patients remained in each length group, and 202 patients remained in each diameter group. Patient characteristics between groups were well balanced.

**Table 1:** Patient characteristics grouped by tumor length (n = 682)

Characteristic	Tumor length			Tumor length (after matching)		
	≤6 cm (n = 399)	>6 cm (n = 283)	*P* value	≤6 cm (n = 212)	>6 cm (n = 212)	*P* value
Age (years)						
≤65 years	186 (46.6%)	161 (56.9%)	.008	119 (56.1%)	103 (48.6%)	.120
>65 years	213 (53.4%)	122 (43.1%)		93 (43.9%)	109 (51.4%)	
Sex						
Male	266 (66.7%)	230 (81.3%)	.000	167 (78.8%)	166 (78.3%)	.906
Female	133 (33.3%)	53 (18.7%)		45 (21.2%)	46 (21.7%)	
N stage						
N0	120 (30.1%)	24 (8.5%)	.000	32 (15.1%)	24 (11.3%)	.789
N1	267 (66.9%)	233 (82.3%)		171 (80.7%)	180 (84.9%)	
N2	10 (2.5%)	23 (8.1%)		7 (3.3%)	7 (3.3%)	
N3	2 (0.5%)	3 (1.1%)		2 (0.9%)	1 (0.5%)	
Chemotherapy						
Yes	258 (64.7%)	199 (70.3%)	.122	143 (67.5%)	143 (67.5%)	1.000
No	141 (35.3%)	84 (29.7%)		69 (32.5%)	69 (32.5%)	
Tumor location						
Cervical/upper	135 (33.8%)	61 (21.6%)	.002	54 (25.5%)	48 (22.6%)	.274
Middle	220 (55.1%)	180 (63.6%)		122 (57.5%)	131 (61.8%)	
Lower	44 (11.0%)	42 (14.8%)		36 (17.0%)	33 (15.6%)	

**Table 2:** Patient characteristics grouped by tumor diameter (n = 682)

Characteristic	Tumor diameter			Tumor diameter (after matching)		
	≤3.5 cm (n = 358)	>3.5 cm (n = 324)	*P* value	≤3.5 cm (n = 202)	>3.5 cm (n = 202)	*P* value
Age (years)						
≤65 years	188 (52.5%)	159 (49.1%)	.375	92 (45.5%)	95 (47.0%)	.765
>65 years	170 (47.5%)	165 (50.9%)		110 (54.5%)	107 (53.0%)	
Sex						
Male	244 (68.2%)	252 (77.8%)	.005	156 (77.2%)	143 (70.8%)	.140
Female	114 (31.8%)	72 (22.2%)		46 (22.8%)	59 (29.2%)	
N stage						
N0	120 (33.5%)	24 (7.4%)	.000	28 (13.9%)	24 (11.9%)	.593
N1	226 (63.2%)	274 (84.6%)		166 (82.2%)	172 (85.1%)	
N2	10 (2.8%)	23 (7.1%)		8 (4.0%)	5 (2.5%)	
N3	2 (0.6%)	3 (0.9%)		0 (0.0%)	1 (0.5%)	
Chemotherapy						
Yes	228 (63.7%)	229 (70.7%)	.052	143 (70.8%)	131 (64.9%)	.201
No	130 (36.3%)	95 (29.3%)		59 (29.2%)	71 (35.1%)	
Tumor location						
Cervical/upper	140 (39.1%)	56 (17.3%)	.000	52 (25.8%)	52 (25.8%)	.431
Middle	177 (49.4%)	223 (68.8%)		119 (58.9%)	118 (58.4%)	
Lower	41 (11.5%)	45 (13.9%)		31 (15.3%)	32 (15.8%)	

### 3.2 Survival with respect to tumor length

**Figure 1:**
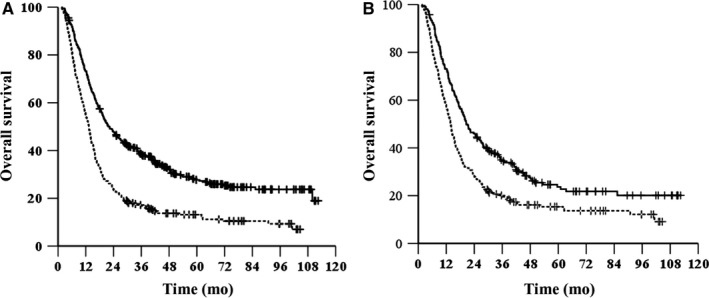
Kaplan–Meier survival curves of OS for patients grouped by length before (A) and after (B) propensity score matching. —, length ≤ 6 cm; ‐‐‐, length > 6 cm

**Figure 2:**
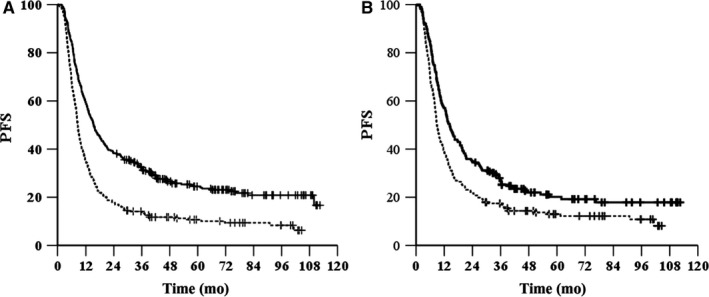
Kaplan‐Meier survival curves of PFS for patients grouped by length before (A) and after (B) propensity score matching. —, length ≤ 6 cm; ‐‐‐, length > 6 cm

The median OS time was 22.2 months (95% CI, 18.5‐25.8 months) in the tumor length ≤ 6 cm group and 13.4 months (95% CI, 12.1‐14.6 months) in the tumor length > 6 cm group (*χ* ^2^ = 50.654, *P* < .001, HR = 1.85, 95% CI, 1.558‐2.198, Figure 1A). The 1‐, 3‐ and 5‐year OS rates were 73.3%, 38.6%, and 27.7%, respectively, in the tumor length ≤ 6 cm group and 54.8%, 17.2%, and 13.1%, respectively, in the tumor length > 6 cm group. The median PFS was 15.4 months (95% CI, 13.1‐17.7 months) in the tumor length ≤ 6 cm group and 8.5 months (95% CI, 7.8‐9.3 months) in the tumor length > 6 cm group (*χ* ^2^ = 48.362, *P* < .001, HR = 1.803, 95% CI, 1.523‐2.134, Figure 2A). The 1‐, 3‐, and 5‐year PFS rates were 59.4%, 32.4% and 24.5%, respectively, in the tumor length ≤ 6 cm group and 35%, 14.1% and 10.7%, respectively, in the tumor length > 6 cm group.

After matching (212 patients in each group), the median OS was 20.6 months (95% CI, 16.7‐24.4 months) in the tumor length ≤ 6 cm group and 13.8 months (95% CI, 12.4‐15.2 months) in the tumor length > 6 cm group (*χ* ^2^ = 16.719, *P* < .001, HR = 1.56, 95% CI, 1.258‐1.934, Figure 1B). The median PFS was 14.3 months (95% CI 12‐16.5 months) in the tumor length ≤ 6 cm group and 8.8 months (95% CI, 7.8‐9.8 months) in the tumor length > 6 cm group (*χ* ^2^ = 22.132, *P* < .001, HR = 1.475, 95% CI, 1.196‐1.819, Figure 2B).

### 3.3 Survival with respect to tumor diameter

**Figure 3:**
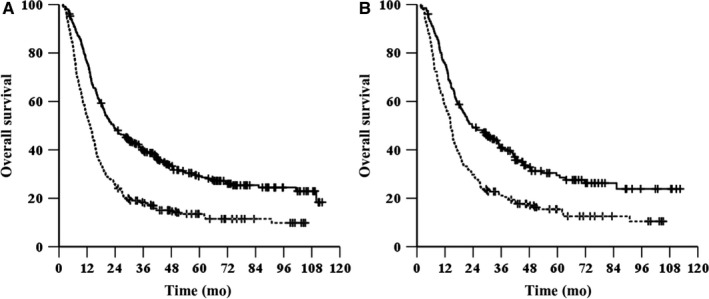
Kaplan‐Meier survival curves of OS for patients grouped by diameter before (A) and after (B) propensity score matching. —, diameter ≤ 3.5 cm; ‐‐‐, diameter > 3.5 cm

**Figure 4:**
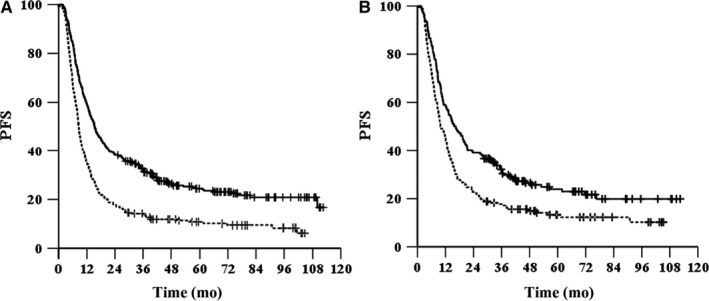
Kaplan‐Meier survival curves of PFS for patients grouped by diameter before (A) and after (B) propensity score matching. —, diameter ≤ 3.5 cm; ‐‐‐, diameter > 3.5 cm

The median OS was 23.3 months (95% CI, 18.5‐28.2 months) in the tumor diameter ≤ 3.5 cm group and 13.3 months (95% CI, 11.8‐14.8 months) in the tumor diameter > 3.5 cm group (*χ* ^2^ = 52.884, *P* < .001, HR = 1.875, 95% CI, 1.578‐2.227, Figure 3A). The 1‐, 3‐ and 5‐year OS rates were 76.1%, 40%, and 29.1%, respectively, in the tumor diameter ≤ 3.5 cm group and 54%, 18.4%, and 13.5%, respectively, in the tumor diameter > 3.5 cm group. The median PFS was 15.9 months (95% CI, 13‐18.8 months) in the tumor diameter ≤ 3.5 cm group and 8.8 months (95% CI, 7.9‐9.6 months) in the tumor diameter > 3.5 cm group (*χ* ^2^ = 44.062, *P* < .001, HR = 1.755, 95% CI, 1.483‐2.077, Figure 4A). The 1‐, 3‐, and 5‐year PFS rates were 59.2%, 33.9% and 25.4%, respectively, in the tumor diameter ≤ 3.5 cm group and 38.3%, 14.7%, and 11.4%, respectively, in the tumor diameter > 3.5 cm group.

After matching (202 patients in each group), the median OS was 23.5 months (95% CI, 16.1‐30.9) in the tumor diameter ≤ 3.5 cm group and 14.4 months (95% CI, 13‐15.8 months) in the tumor diameter > 3.5 cm group (*χ* ^2^ = 22.733, *P* < .001, HR = 1.719, 95% CI, 1.372‐2.154, Figure 3B). The median PFS was 15.8 months (95% CI, 12‐19.5 months) in the tumor diameter ≤ 3.5 cm group and 9.8 months (95% CI, 8‐11.7 months) in the tumor diameter > 3.5 cm group (*χ* ^2^ = 14.997, *P* < .001, HR = 1.535, 95% CI, 1.233‐1.91, Figure 4B).

### 3.4 Prognostic factor analysis

Univariate analyses were performed to assess the predictive capability of each variable assessed. As shown in Table 3, sex, tumor location, tumor length, tumor diameter, chemotherapy and N stage were found to be significantly associated with survival. All factors influencing prognosis were included in the multivariate analysis to determine the independent prognostic factors for patients with ESCC treated with (chemo)radiotherapy. As shown in Table 4, sex, tumor location, tumor length, tumor diameter, chemotherapy and N stage could potentially serve as independent prognostic factors (*P* < .05).

**Table 3:** Univariate analysis of prognostic factors for patients with esophageal squamous cell cancer

Variable	Hazard ratio for OS		Hazard ratio for PFS	
	*P* value	HR (95% CI)	*P* value	HR (95% CI)
Sex (male/female)	.000	0.699 (0.573‐0.854)	.000	0.699 (0.576 −0.849)
Age (≤65/>65 years)	.964	1.004 (0.847‐1.191)	.958	1.005 (0.850‐1.187)
Diameter (≤3.5/>3.5 cm)	.000	1.875 (1.578‐2.227)	.000	1.755 (1.483‐2.077)
Length (≤6/>6 cm)	.000	1.850 (1.558‐2.198)	.000	1.803 (1.523‐2.134)
Chemotherapy (yes/no)	.048	0.836 (0.700‐0.999)	.155	0.881 (0.741‐1.029)
Tumor site (cervical + upper/middle/lower)	.000	1.308 (1.143‐1.497)	.000	1.314 (1.152‐1.500)
N stage (N0/N1/N2/N3)	.000	1.876 (1.587‐1.218)	.000	1.790 (1.529‐2.095)

**Table 4:** Multivariate analysis of prognostic factors for patients with esophageal squamous cell cancer

Variable	Hazard ratio for OS		Hazard ratio for PFS	
	*P* value	HR (95% CI)	*P* value	HR (95% CI)
Sex (male/female)	.005	0.747 (0.608‐0.918)	.019	0.789 (0.647‐0.961)
Diameter (≤3.5/>3.5 cm)	.004	1.3.62 (1.105‐1.679)	.029	1.260 (1.025‐1.548)
Length (≤6/>6 cm)	.004	1.351 (1.100‐1.658)	.005	1.339 (1.093‐1.640)
Chemotherapy (yes/no)	.002	0.752 (0.626‐0.904)	—	
Tumor site (cervical + upper/middle/lower)	.128	1.117 (0.969‐1.288)	.019	1.178 (1.027‐1.352)
N stage (N0/N1/N2/N3)	.000	1.611 (1.342‐1.934)	.000	1.537 (1.296‐1.822)

## 4 DISCUSSION

The current clinical staging system for esophageal cancer is not suitable for patients treated with (chemo)radiotherapy due to its prognostic inaccuracy and difficulty in practice.^4^, ^5^ In 2010, the Chinese clinical staging expert group proposed that the tumor length determined by barium esophagography and the tumor diameter determined by the maximum esophageal diameter shown by CT be considered criteria for the nonsurgical T staging of esophageal cancer.^22^ However, this guideline remains in draft form and has not since been updated and lacks support from high‐level evidence. Recently, an increasing number of scholars have concentrated on the nonsurgical staging of ESCC.^20^, ^23^ To contribute the data and experience from our center, we analyzed 682 ESCC patients treated with (chemo)radiotherapy in this study. The sample size is relatively sufficient, and PMS analysis made the results more reliable. We confirmed the independent prognostic value of tumor length and diameter and suggest that these two indicators be considered when developing nonsurgical staging for ESCC.

In 2002, Eloubeidi et al identified 10 441 esophageal cancer patients from the National Cancer Institute Surveillance, Epidemiology, and End Results (SEER) Program and found that tumor length was an independent predictor of mortality in patients with localized disease.^10^ Similar results have been found in most successively published studies.^11^, ^12^, ^13^, ^14^, ^15^, ^16^, ^24^ Some studies have even found that a longer tumor length is associated with higher T stage and N stage.^11^, ^12^ Additionally, three recently published studies found that larger tumor lengths were associated with pathologic upstaging, as clinical staging for T2N0 disease remains highly inaccurate.^25^, ^26^, ^27^ This growing evidence indicates that tumor length is valuable for staging, but most studies have been based on surgical patients. Chang's study prospectively detected tumor length by mini‐EUS in ESCC patients treated with concurrent chemoradiotherapy and found that a tumor length ≥ 6 cm was a negative predictor of treatment response and survival.^28^ A study from MD Anderson Cancer Center focused on tumor recurrence found that a primary tumor length > 5 cm was the only adverse independent prognostic factor in ESCC after multivariate analysis.^29^ In our study, we found that the tumor length ≤ 6 cm group had a significantly better PFS/OS than the tumor length > 6 cm group. Similar results were obtained even after 1:1 PSM. Multivariate analysis also showed that tumor length was an independent prognostic factor affecting the prognosis of ESCC patients.

It should be noted that the optimal cut‐off value of tumor length varies among studies,^10^, ^11^, ^12^, ^13^, ^14^, ^15^, ^16^, ^17^, ^18^, ^19^, ^20^, ^28^, ^29^ ranging from 2 to 7 cm, with 3 cm being the most common cut‐off point. The main reason for this discrepancy is the heterogeneity among studies, such as treatment, stage of disease, detection modality, sample size and method of generating the cut‐off point, and it also partly explains why several studies have found negative results between tumor length and survival.^17^, ^18^, ^19^ In our study, most patients had locally advanced lesions and were thus excluded from curative surgery; thus, the cut‐off value was 6 cm, which is much longer than that used in other surgical studies^11^, ^13^, ^14^, ^15^ but similar to that used in Kim's,^19^ Chang's^28^ and Xi's^29^ studies. The optimal cut‐off point for tumor length may need further refinement, which will require further assessment with larger prospective datasets.

Tumor length can be detected by several methods. The endoscope is a useful and visual examination but is limited in stenotic tumors. Because the target area is countered mostly in CT images, some scholars prefer the use of CT to indicate tumor length with a standard wall thickness of >5 mm.^20^, ^30^ This method is also unreliable because nonspecific changes, such as inflammatory edema, may occur around the lesion, especially in the lower esophagus.^31^ Based on our center's practice, esophageal length is indicated mostly by esophagography^22^ and partly referring to the endoscope and CT image. Therefore, we used the former for analysis in this study. Esophagography is usually the initial examination for esophageal cancer patients. It can reflect the mucosal irregularity along the axis of the esophagus at the level of the tumor and identify a polypoidal, ulcerous or stenotic tumor. It also offers additional diagnostic information, such as the location of the tumor and the presence of diverticulum and fistula.^32^ However, the best modality to detect tumor length requires further investigation.

We also found that tumor diameter is an independent prognostic factor for ESCC patients treated with (chemo)radiotherapy. A similar result was found after PSM. In a study by Cai,^20^ they analyzed 324 surgical ESCC patients and confirmed the prognostic value of tumor diameter (described as the maximum long diameter in their study) by CT. They recommended prognostic predictions according to tumor diameters of <28.7 mm, 28.7‐34.6 mm, 34.6‐41.4 mm and > 41.4 mm. Chen et al^18^ also found similar results to our study. They retrospectively analyzed 153 ESCC patients treated with radiation and found that tumor diameter is one of the best predictors for survival using a cut‐off value of 2 cm. However, they emphasized that the tumor diameter should be measured by the anterior‐posterior tumor dimension, which differs from the method used in our study.

There is no doubt that the invasion depth into the esophageal wall is a vital prognostic factor for esophageal cancer. It would be interesting to know whether the maximum transverse diameter can somehow reflect the invasion depth because they are present on the same dimension. In Li's study,^30^ they found that the maximal tumor thickness (consistent with diameter) based on CT increased with advancing T category. However, in Cai's study,^20^ the larger tumor diameter did not significantly correspond to advanced pathologic T stage. The different cut‐off points and analytical methods may have caused discordance.

Gross tumor volume has also been suggested to guide the T stage.^18^, ^23^, ^30^ Reasonable stratification of gross tumor volume measured with CT could distinguish the prognosis of esophageal cancer patients treated with (chemo)radiotherapy.^18^, ^23^ In fact, tumor length combined with diameter can reflect gross volume to a certain extent but still differs from it. The first two parameters represent initial tumor features, while volume is based on a secondary calculation. However, it is reasonable to combine tumor length and diameter to develop the nonsurgical T staging system based on our results. The optimal combination needs further study.

This study has some drawbacks. First, our results were limited because the data were obtained from a single‐center setting and the study was retrospective in nature. Second, some of the patients received radiation alone, which is not a standard curative treatment for nonsurgical cases. Third, we provide only a referential cut‐off point for tumor length and diameter but have not generated definite stratifications of the T category. A fistula or deep ulcer indicated by esophagography and an adjacent invasion indicated by CT may help identify an advanced T stage (probably T4). Further work will address the optimization grouping based on the findings of this study.

## 5 CONCLUSION

The results of our study suggest that esophageal tumor length and diameter determined by radiography are valuable prognostic factors for ESCC patients undergoing definitive (chemo)radiotherapy. Because tumor depth is difficult to detect without surgery, this study offers two potential indicators to develop a nonsurgical T staging system. Further work in this area is needed.

## CONFLICT OF INTEREST

Conflict of interest relevant to this article was not reported.

## Data Availability

The data that support the findings of this study are available from the corresponding author upon reasonable request.
