# Supplementary material for: Prognostic value of tumor length and diameter for esophageal squamous cell cancer patients treated with definitive (chemo)radiotherapy: Potential indicators for nonsurgical T staging
Source: Cancer Med. 2019 Sep 4;8(14):6326–34. doi: 10.1002/cam4.2532 (PMC6797578; doi:10.1002/cam4.2532)
Supplement: Supplementary file 1 [file CAM4-8-6326-s001.docx]

[Supplementary](javascript:void(0);) table Sensitivity and specificity value of tumor diameter calculated by ROC analysis

| Threshold | sensitivity | specificity | sensitivity + specificity |
| --- | --- | --- | --- |
| 0.400 | 1.000 | 0.000 | 1.000 |
| 1.450 | 0.996 | 0.000 | 0.996 |
| 1.550 | 0.993 | 0.024 | 1.017 |
| 1.650 | 0.993 | 0.031 | 1.024 |
| 1.750 | 0.987 | 0.031 | 1.018 |
| 1.850 | 0.984 | 0.047 | 1.031 |
| 1.950 | 0.982 | 0.079 | 1.061 |
| 2.050 | 0.973 | 0.087 | 1.060 |
| 2.150 | 0.968 | 0.102 | 1.070 |
| 2.250 | 0.950 | 0.173 | 1.123 |
| 2.350 | 0.935 | 0.189 | 1.124 |
| 2.450 | 0.892 | 0.252 | 1.144 |
| 2.550 | 0.879 | 0.307 | 1.186 |
| 2.650 | 0.849 | 0.362 | 1.211 |
| 2.750 | 0.840 | 0.394 | 1.234 |
| 2.850 | 0.793 | 0.449 | 1.242 |
| 2.950 | 0.759 | 0.504 | 1.263 |
| 3.050 | 0.726 | 0.559 | 1.285 |
| 3.150 | 0.699 | 0.598 | 1.297 |
| 3.250 | 0.656 | 0.638 | 1.294 |
| 3.350 | 0.595 | 0.661 | 1.256 |
| 3.450 | 0.564 | 0.693 | 1.257 |
| 3.550 | 0.515 | 0.701 | 1.216 |
| 3.650 | 0.476 | 0.732 | 1.208 |
| 3.750 | 0.445 | 0.772 | 1.217 |
| 3.850 | 0.409 | 0.795 | 1.204 |
| 3.950 | 0.391 | 0.803 | 1.194 |
| 4.050 | 0.386 | 0.811 | 1.197 |
| 4.150 | 0.332 | 0.835 | 1.167 |
| 4.250 | 0.252 | 0.866 | 1.118 |
| 4.350 | 0.227 | 0.882 | 1.109 |
| 4.450 | 0.200 | 0.906 | 1.106 |
| 4.550 | 0.164 | 0.929 | 1.093 |
| 4.650 | 0.139 | 0.937 | 1.076 |
| 4.750 | 0.119 | 0.953 | 1.072 |
| 4.850 | 0.095 | 0.961 | 1.056 |
| 4.950 | 0.090 | 0.961 | 1.051 |
| 5.050 | 0.074 | 0.969 | 1.043 |
| 5.150 | 0.054 | 0.969 | 1.023 |
| 5.250 | 0.047 | 0.984 | 1.031 |
| 5.350 | 0.038 | 0.984 | 1.022 |
| 5.450 | 0.034 | 0.984 | 1.018 |
| 5.550 | 0.031 | 0.992 | 1.023 |
| 5.650 | 0.020 | 0.992 | 1.012 |
| 5.750 | 0.013 | 0.992 | 1.005 |
| 5.850 | 0.009 | 0.992 | 1.001 |
| 6.050 | 0.007 | 0.992 | 0.999 |
| 6.350 | 0.002 | 0.992 | 0.994 |
| 6.550 | 0.002 | 1.000 | 1.002 |
| 7.600 | 0.000 | 1.000 | 1.000 |

[Supplementary](javascript:void(0);) table Sensitivity and specificity value of tumor length calculated by ROC analysis

| Threshold | sensitivity | specificity | sensitivity + specificity |
| --- | --- | --- | --- |
| 0.000 | 1.000 | 0.000 | 1.000 |
| 1.200 | 0.998 | 0.008 | 1.006 |
| 1.450 | 0.996 | 0.008 | 1.004 |
| 1.600 | 0.995 | 0.008 | 1.003 |
| 1.850 | 0.993 | 0.016 | 1.009 |
| 2.200 | 0.991 | 0.031 | 1.022 |
| 2.450 | 0.989 | 0.055 | 1.044 |
| 2.650 | 0.978 | 0.071 | 1.049 |
| 2.850 | 0.977 | 0.071 | 1.048 |
| 2.950 | 0.975 | 0.071 | 1.046 |
| 3.050 | 0.964 | 0.110 | 1.074 |
| 3.150 | 0.960 | 0.110 | 1.070 |
| 3.250 | 0.951 | 0.134 | 1.085 |
| 3.400 | 0.946 | 0.134 | 1.080 |
| 3.550 | 0.905 | 0.181 | 1.086 |
| 3.650 | 0.901 | 0.189 | 1.090 |
| 3.750 | 0.897 | 0.197 | 1.094 |
| 3.850 | 0.894 | 0.213 | 1.107 |
| 3.950 | 0.886 | 0.213 | 1.099 |
| 4.100 | 0.832 | 0.323 | 1.155 |
| 4.250 | 0.822 | 0.331 | 1.153 |
| 4.350 | 0.818 | 0.346 | 1.164 |
| 4.450 | 0.811 | 0.346 | 1.157 |
| 4.550 | 0.771 | 0.386 | 1.157 |
| 4.650 | 0.766 | 0.409 | 1.175 |
| 4.800 | 0.757 | 0.425 | 1.182 |
| 4.950 | 0.753 | 0.425 | 1.178 |
| 5.050 | 0.715 | 0.449 | 1.164 |
| 5.150 | 0.705 | 0.480 | 1.185 |
| 5.250 | 0.679 | 0.504 | 1.183 |
| 5.350 | 0.668 | 0.512 | 1.180 |
| 5.450 | 0.661 | 0.535 | 1.196 |
| 5.550 | 0.595 | 0.638 | 1.233 |
| 5.650 | 0.578 | 0.669 | 1.247 |
| 5.750 | 0.571 | 0.677 | 1.248 |
| 5.850 | 0.566 | 0.701 | 1.267 |
| 5.950 | 0.562 | 0.701 | 1.263 |
| 6.050 | 0.459 | 0.780 | 1.239 |
| 6.150 | 0.458 | 0.780 | 1.238 |
| 6.250 | 0.443 | 0.780 | 1.223 |
| 6.350 | 0.438 | 0.780 | 1.218 |
| 6.450 | 0.432 | 0.780 | 1.212 |
| 6.550 | 0.375 | 0.819 | 1.194 |
| 6.650 | 0.371 | 0.819 | 1.190 |
| 6.750 | 0.364 | 0.819 | 1.183 |
| 6.850 | 0.351 | 0.819 | 1.170 |
| 6.950 | 0.341 | 0.827 | 1.168 |
| 7.050 | 0.324 | 0.843 | 1.167 |
| 7.150 | 0.314 | 0.843 | 1.157 |
| 7.250 | 0.290 | 0.858 | 1.148 |
| 7.350 | 0.279 | 0.134 | 0.413 |
| 7.450 | 0.274 | 0.134 | 0.408 |
| 7.550 | 0.220 | 0.102 | 0.322 |
| 7.650 | 0.218 | 0.087 | 0.305 |
| 7.750 | 0.216 | 0.087 | 0.303 |
| 7.850 | 0.214 | 0.079 | 0.293 |
| 7.950 | 0.211 | 0.079 | 0.290 |
| 8.050 | 0.146 | 0.063 | 0.209 |
| 8.200 | 0.142 | 0.063 | 0.205 |
| 8.350 | 0.139 | 0.063 | 0.202 |
| 8.450 | 0.137 | 0.063 | 0.200 |
| 8.600 | 0.115 | 0.039 | 0.155 |
| 8.750 | 0.114 | 0.039 | 0.153 |
| 8.900 | 0.112 | 0.031 | 0.143 |
| 9.050 | 0.086 | 0.024 | 0.110 |
| 9.150 | 0.081 | 0.024 | 0.105 |
| 9.250 | 0.079 | 0.024 | 0.103 |
| 9.400 | 0.077 | 0.024 | 0.101 |
| 9.600 | 0.072 | 0.024 | 0.096 |
| 9.750 | 0.070 | 0.024 | 0.094 |
| 9.900 | 0.067 | 0.024 | 0.090 |
| 10.250 | 0.047 | 0.016 | 0.063 |
| 10.750 | 0.041 | 0.016 | 0.057 |
| 11.200 | 0.032 | 0.016 | 0.048 |
| 11.700 | 0.031 | 0.016 | 0.046 |
| 12.100 | 0.018 | 0.008 | 0.026 |
| 12.350 | 0.016 | 0.008 | 0.024 |
| 12.650 | 0.013 | 0.008 | 0.020 |
| 12.900 | 0.011 | 0.008 | 0.019 |
| 13.500 | 0.009 | 0.008 | 0.017 |
| 14.600 | 0.007 | 0.008 | 0.015 |
| 16.100 | 0.005 | 0.008 | 0.013 |
| 19.250 | 0.002 | 0.000 | 0.002 |
| 22.500 | 0.000 | 0.000 | 0.000 |
